# Utilization of health facilities and predictors of health-seeking behavior for under-five children with acute diarrhea in slums of Addis Ababa, Ethiopia: a community-based cross-sectional study

**DOI:** 10.1186/s41043-017-0085-1

**Published:** 2017-04-04

**Authors:** Metadel Adane, Bezatu Mengistie, Worku Mulat, Helmut Kloos, Girmay Medhin

**Affiliations:** 1 Ethiopian Institute of Water Resources (EIWR), Addis Ababa University, Addis Ababa, Ethiopia; 2 College of Health and Medical Sciences, Haramaya University, Haramaya, Ethiopia; 3 Department of Civil and Environmental Engineering, University of Connecticut, Storrs, USA; 4 Department of Epidemiology and Biostatistics, University of California, San Francisco, USA; 5 Aklilu Lemma Institute of Pathobiology, Addis Ababa University, Addis Ababa, Ethiopia

**Keywords:** Andersen behavioral model, Enabling factor, Health-seeking behavior, Health facilities, Mothers/caregivers, Need factor, Predisposing factor, Under-five children

## Abstract

**Background:**

Information on health-seeking behavior and utilization of health facilities in slums of Addis Ababa is scarce, impeding the implementation of effective interventions. The purpose of this study is to assess the status of health facilities utilization and predictors for health-seeking behavior of mothers/caregivers of under-five children with acute diarrhea in slums of Addis Ababa, Ethiopia.

**Methods:**

A community-based cross-sectional study design was employed in five rounds of surveys in seven *kebeles* in slums of Addis Ababa among 472 mothers/caregivers of 472 under-five children with acute diarrhea in reference to Andersen’s behavioral model. Data were entered into EpiData Version 3.1 and analyzed using STATA Version 14.0. Descriptive statistics were used to examine patterns of health facilities utilization and multivariable logistic regression analysis was applied to identify predictors associated with health-seeking behavior.

**Results:**

Most mothers/caregivers (70.8%) sought care either at home (14.2%) or health facilities (56.6%), whereas 29.2% reported that they did not seek any care. Of those who consulted health facilities, government health facilities (76.9%) were more utilized than private (18.0%) and informal (5.1%) health facilities. Nearly all (93.9%) of the mothers/caregivers using government health facilities used health centers, and of those who took their children to private health facilities (60.9%) used clinics and 26.1% used pharmacies/drug vendors. Mothers/caregivers visiting health facilities obtained mainly oral rehydration salt (ORS) (39.8%) and home-recommended fluids (HRF) (40.3%), but few of them (11.9%) obtained ORS plus zinc supplementation. Predisposing factors of literacy of mothers/caregivers (adjusted odds ratio (AOR) = 2.4; 95% CI 1.4–4.1) and occupation (AOR = 2.6; 95% CI 1.5–4.6), the enabling factors of households monthly income of 50 United States Dollars (US$) and above (AOR = 2.9; 95% CI 1.5–5.6) and availability of nearest health facilities within 15 min walking distance (AOR = 3.3; 95% CI 1.7–6.6), and the need factors of recognizing danger signs of fever (AOR = 4.3; 95% CI 2.4–7.6) and vomiting (AOR = 3.3; 95% CI 1.8–5.9) were significantly associated with health-seeking behavior.

**Conclusions:**

Increasing the proximity of health facilities in slums and health education and socioeconomic development programs targeting illiterate mothers/caregivers and poor households may promote and increase health-seeking behavior and the accessibility of health facilities for the treatment of acute diarrhea in under-five children in Addis Ababa slums.

## Background

Sub-Saharan Africa has made the least progress in the reduction of infant and child mortality [1]. The two leading causes of mortality among children under-five years of age in sub-Saharan Africa are pneumonia and diarrhea, accounting for 18% and 15%, respectively [2]. Appropriate health-seeking behavior can reduce child morbidity and mortality due to diarrhea [3] and is vital for integrated management of childhood illness [4, 5] and child survival [6]. Based on Andersen’s behavioral model, predisposing, enabling, and need factors at the individual and community levels are instrumental for increasing health-seeking behavior and health facilities utilization [7–9]. Individual and community level factors also determine the occurrence and outcome of diarrhea [8, 10].

Most studies using Andersen’s behavioral model used secondary data with different categorizations of variables, indicating that primary studies are needed to identify predictors of health-seeking behavior [11]. Studies from various countries suggest that health-seeking behavior for childhood illnesses is often inappropriate and health facilities are under-utilized, particularly by the poor [12–16]. The 2011 citywide Demographic Health Survey in Addis Ababa indicated that less than half (47.2%) of mothers/caregivers sought treatment for diarrhea at health facilities [17]. An understanding of health-seeking behavior is vital for the rational planning and evaluation of health services. However, in Ethiopia's urban, including slums of Addis Ababa, there is limited information on health facility utilization and predictors for health-seeking behavior for under-five children with acute diarrhea. In the current study, we adapted the Andersen behavioral model [9] as a conceptual framework to identify predictors needed to operationalize the need, predisposing, and enabling factors for enhancing health-seeking behavior for under-five children with acute diarrhea in slums of Addis Ababa.

The results may assist health policy makers, planners, non-governmental organizations, and other bodies interested in reducing child mortality and morbidity through promoting health-seeking in health facilities in slums of Addis Ababa and Ethiopia's other urban slums.

## Methods

### Study setting

The study was conducted in slum areas of Addis Ababa, the capital city of Ethiopia. Four slum *kebeles* in Gullele Sub-City’s District (W*oreda*) 01 and three slum *kebeles* in Lideta Sub-City’s District 05 were included in the study. There is one governmental health center in District 01, in Gullele Sub-City, but none in District 05 in Lideta Sub-City. Thus, residents of District 05 have to be served by other District health facilities.

Addis Ababa’s population was projected to be 3.27 million in 2014/2015, 47.4% of them males and 52.6% females [18]. According to the 2015 report by the District administration, there were 1453 legally registered under-five children in District 01, in Gullele Sub-City, and 1321 in District 05 in Lideta Sub-City. Ethiopia has both public and private sector health facilities. Private sector health facilities (clinics, hospitals, pharmacies, and/or drug vendors) provide mainly curative services, whereas public sector health facilities provide both curative and all preventive services. A considerable number of traditional healers also continue to operate in the informal health facility sector [19].

### Study design, period, and populations

Five rounds of a community-based cross-sectional survey (one round during a case–control study and four rounds when a longitudinal study conducted) within periods of October 2014 (first round), November to December 2014 (second round), January 2015 (third round), April 2015 (fourth round), and July 2015 (fifth round) was employed. The source populations were all under-five children who had acute diarrhea during the study period in slums of Addis Ababa, whereas the study populations were under-five children with acute diarrhea that lived in the seven slum *kebeles* during the survey period whose mothers/caregivers consented to their participation in the study.

### Sample size calculation

For sample size estimation, we assumed a 95% confidence interval (CI), a margin of error of 5%, a proportion of 47.2% for health-seeking in one or more health facilities in Addis Ababa [17], and a design effect of 1.5. We used design effect since the multistage sampling method was employed. A minimum adequate sample size was calculated based on the statistical estimation method of Kelsey et al. [20]. Since the source population was estimated less than 10,000; sample size correction was performed. Then, 10% non-response rate was added to obtain the adequate sample size of 429. To satisfy the minimum adequate sample size criteria, the study included all 472 under-five children with acute diarrhea during the five rounds of survey period based on the inclusion and exclusion criteria.

### Inclusion and exclusion criteria

If the same under-five children had more than one episode, we only considered the first acute diarrheal case and excluded the repeated case for the study. When the same mother/caregiver had another child with diarrhea, it was excluded for the study if that child was not part of the longitudinal follow-up study because we used systematic sampling technique from the initial recruitment of children for the follow-up study. During the case–control study, when there was more than one child with acute diarrhea per household, we randomly choose one of the children before interviewing the mother/caregiver. Under-five children with bloody and persistent diarrhea were also excluded.

### Operational definitions

Health-seeking behavior was defined as diarrheal management practices by mothers/caregivers of under-five children with acute diarrhea for the treatment and recovery of the child from acute diarrhea within 2 weeks prior to data collection. Acute diarrhea was identified using World Health Organization (WHO) signs and symptoms for diarrhea by questioning the mothers/caregivers first about signs and symptoms of diarrhea such as consistency of bowel movements, fever, vomiting, blood in stool, mucus in stool, watery stool, and the frequency of abnormal stool 2 weeks prior to data enumeration. We defined acute diarrhea following the WHO protocol [21] as the passage of three or more abnormally loose watery or liquid stools over a 24 h period. However, the WHO protocol did not specify the recall period. We used the 2 week recall period as specified in World Gastroenterology Organization global guidelines for acute diarrhea survey [22]. We therefore define acute diarrhea as the passage of three or more abnormally loose watery or liquid stools over a 24 h period within 2 weeks prior to the data collection.

### Explanatory variables

**Fig. 1:**
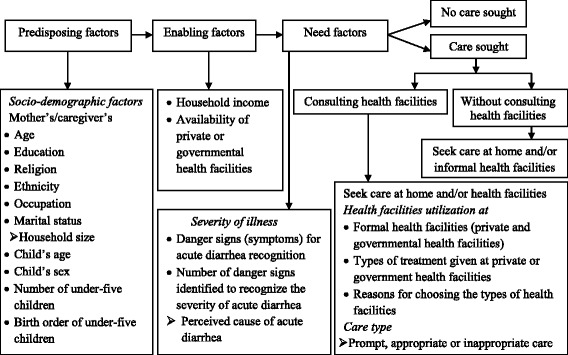
Conceptual framework for health-seeking behavior modified from Andersen’s behavioral model

We selected explanatory variables based on Andersen’s behavioral model [9] (Fig. 1), which is widely accepted and used to study the predictors of health-seeking behavior and health facility utilization for various diseases, including childhood diarrhea [23, 24]. Predisposing factors considered for analysis were mothers/caregivers education as a dichotomous variable, age in three categories, ethnicity in five categories, religion as a dichotomous variable, marital status classified as married or currently not married, occupation as binary (housewife versus not a housewife), household size classified into two categories (2–5, 6 or more), age of under-five children was classified into five categories (0–11, 12–23, 24–35, 36–47, and 48–59 months), number of under-five children in the house was categorized into two (1 child, 2 children or more), and birth order of under-five children was categorized into four (first, second, third, and fourth and above).

Enabling factors were monthly household income and availability of nearest health facilities within 15 min walking distance. Of the need factors (severity of illness), we included perceived cause of acute diarrhea, recognition of acute diarrhea danger signs, and number of danger signs (1, 2, 3 and 4 or more). Information was obtained from mothers/caregivers on the types of health facilities used and reasons for these choices. Weaning of food and liquid status during acute diarrheal illness and the types of treatment given at government and private health facilities were also studied.

### Household survey, data quality control, and data management

Data were collected using a pretested structured questionnaire. The content of the questionnaire covered predisposing, enabling, and need factors based on the adapted Andersen behavioral model conceptual framework (Fig. 1).

The questionnaire was prepared in English and translated into Amharic (local language). To maintain the consistency of the content, it was translated back into English and necessary adjustments were made before the translation back into Amharic for use in the field. The structured questionnaire was pretested in 10% of the sample size in one randomly selected slum *kebele* out of the studied *kebeles* and amended where necessary.

The principal investigator provided 2 days of training for seven field data enumerators and two supervisors about the administration of each question and ethical principles. The data collectors were female nurses and environmental health professionals with BSc degrees or diplomas , whereas the supervisors were public health professionals with masters of public health degrees. The primary respondents were the mothers of the under-five-year-old children who had acute diarrhea during the survey period. Where a mother was not a caregiver, another primary caregiver was interviewed. Ten percent of the study participants were randomly selected and re-interviewed by another interviewer to check reliability of the information entered by different interviewers. The completeness of the questionnaires was checked every day in the field by supervisors. The principal investigator then re-checked the questionnaires for consistency, clarity, and completeness. No substitution was made for mothers/caregivers who were either unavailable or unwilling to participate in the study. If a mother/caregiver was not available at the time of the survey, the data collectors scheduled another visit for the same or the next day. If not available again, the household was considered as non-respondent.

The collected data were computerized using EpiData Version 3.1 (EpiData Associations, Odense, Denmark) and then exported to Statistical Package for the Social Sciences (SPSS) Version 24.0 (IBM Corp., Armonk, N.Y., USA) for data cleaning. In order to verify the accuracy of data entries, two generic data verification strategies were employed. As the first step, randomly selected 10% of the questionnaires were thoroughly checked. Following this, descriptive statistics, results from cross-tabulations, and frequency distributions were examined before performing statistical analysis.

### Data analysis

Data were analyzed using STATA Version 14.0 [25]. Proportions for categorical variables and mean (±standard deviations (SD)) for continuous variables were used as descriptive measures. The associations between predisposing (block 1), enabling (block 2), and need (block 3) factors with health-seeking behavior were modeled using bivariate and multivariable logistic regression. Odds ratio with corresponding 95% CI were used to assess the strength of associations of different factors with health-seeking behavior.

Bivariate analysis was employed to identify variables associated with health-seeking behavior without adjusting for potential confounding variables. To reduce excessive numbers of variables, only variables with *p* < 0.3 in bivariate analysis were considered for initial inclusion in the multivariable analysis based on each block [26–28]. Variables with *p* < 0.3 in each model (models 1, 2, and 3) of the multivariable analysis were included in the final model. Then, we estimated the overall effect of the three blocks of variables. Hosmer–Lemeshow statistics was used to test the goodness-of-fit of the model [29]. Variables with *p* < 0.05 in the final model were taken as statistically significant and independently associated with health-seeking behavior.

## Results

### Characteristics of the study participants

A total of 452 mothers/caregivers who had under-five-year-old children with acute diarrhea were enrolled in the survey, with a response rate of 95.8%. Nearly all respondents (98.9%) were biological mothers. The mean household monthly income was 44.7(±25.5) United States Dollars (US$). The mean household size was 5.2(±1.9) persons, and the mean age of children was 27.2(±14.2) months, the mean age of mothers/caregivers was 29.3(±6.1) years, and 330 (73.0%) of them were literate (Table 1).

**Table 1:** Characteristics of mothers/caregivers of under-five children with acute diarrhea in slums of Addis Ababa, October 2014–July 2015

Variables	n (%)	Mean	SD
Health-seeking behavior (N = 452)			
Sought care	320 (70.8)		
No care sought for treating the child	132 (29.2)		
Where sought care given (N = 452)			
At home only without consulting health facilities	64 (14.2)		
At health facilities	256 (56.6)		
Both at home and health facilities^a^	138 (30.5)		
Care type (N = 320)			
Inappropriate care^b^	39 (12.2)		
Prompt care^c^	87 (27.2)		
Monthly household income (US$)		44.7	25.5
Mothers/caregivers age (years)		29.3	6.1
Mothers/caregivers education (N = 452)			
Illiterate	122 (27.0)		
Literate	330 (73.0)		
Household size^d^		5.2	1.9
Child’s age (months)		27.2	14.2
Time of walking to the nearest health facility (minutes)		28	14
Care sought after danger signs of acute diarrhea (days) were recognized		2.3	1.5

N Denominator

^a^Indicates sought care at health facilities and at home with consulting health facilities, it also included multiple response

^b^Care by giving medicine available at home without consulting health facilities, care from informal health facilities (traditional healers), or care from pharmacies or drug vendors

^c^Care that was sought within 24 h after recognition of the presence of diarrhea

^d^The number of persons living together in one house with one household head

### Health-seeking behavior

More than two thirds 320 (70.8%) of the mothers/caregivers sought care for their acute diarrheal under-five-year-old children either at home (14.2%) or health facilities (56.6%), and 29.2% of the mothers/caregivers reported that they did not seek any treatment either at home or in health facilities for the purpose of acute diarrhea treatment. Among mothers/caregivers who sought care, 87 (27.2%) sought care promptly within 24 h after recognizing acute diarrhea and 39 (12.2%) obtained inappropriate care. The mean walking time required by mothers/caregivers to reach the health facilities for seeking care was reportedly 28(±14) min, and the mean number of elapsed days to get care after recognizing the danger sign of acute diarrhea was 2.3(±1.5) days (Table 1).

In bivariate analysis of predisposing factors, mothers/caregivers literacy (crude odds ratio (COR) = 2.8; 95% CI 1.8–4.4), married mother/caregiver (COR = 1.7; 95% CI 1.1–2.8), and occupation (not being a housewife) (COR = 1.6; 95% CI 1.1–2.6) were significantly associated with health-seeking behavior (Table 2). Among enabling factors, having household monthly income of 50 US$ and above, and availability of health facilities within 15 min walking distance were significantly associated with health-seeking behavior. From the need factors, recognizing danger signs of fever, thirst, vomiting, and increasing numbers of danger signs were significantly associated with health-seeking behavior (Table 3).

**Table 2:** Bivariate analysis of predisposing factors with health-seeking behavior in slums of Addis Ababa, October 2014–July 2015

Variables in block 1	Health-seeking		COR (95% CI)
	Sought care (%)	No care sought (%)	
Mothers/caregivers age (years)			
<25	60 (70.6)	25 (29.4)	1
25–34	196 (73.1)	72 (26.9)	1.1 (0.7–1.9)
>34	64 (64.6)	35 (35.4)	0.8 (0.4–1.4)
Mothers/caregivers education			
Literate	254 (77.0)	76 (23.0)	2.8 (1.8–4.4)
Illiterate	66 (54.1)	56 (45.9)	1
Religion of mothers/caregivers			
Christian	288 (70.4)	121 (29.6)	0.8 (0.4–1.7)
Muslim	32 (74.4)	11 (25.6)	1
Ethnicity of mothers/caregivers			
Oromo	74 (73.3)	27 (26.7)	1.1 (0.6–2.0)
Amhara	106 (69.7)	46 (30.3)	0.9 (0.5–1.6)
Tigira	31 (70.5)	13 (29.5)	0.9 (0.4–2.1)
Gurage	40 (67.8)	19 (32.2)	0.8 (0.4–1.7)
Other^a^	69 (71.9)	27 (28.1)	1
Mothers/caregivers occupation			
Not housewife	66 (62.9)	39 (37.1)	1.6 (1.1–2.6)
Housewife	254 (73.2)	93 (26.8)	1
Household size			
2–5 persons	196 (68.3)	91 (31.7)	0.7 (0.5–1.1)
6 or more persons	124 (75.2)	41 (24.8)	1
Marital status of mothers/caregivers			
Married	256 (73.6)	92 (26.4)	1.7 (1.1–2.8)
Currently not married	64 (61.5)	40 (38.5)	1
Number of under-five children			
1 child	231 (69.6)	101 (30.4)	0.8 (0.5–1.3)
2 or more children	89 (74.2)	31 (25.8)	1
Birth order of under-five children			
First	158 (71.2)	64 (28.8)	1.0 (0.5–2.0)
Second	92 (68.7)	42 (31.3)	0.9 (0.4–1.9)
Third	36 (75.0)	12 (25.0)	1.2 (0.5–3.0)
Fourth and above	34 (70.8)	14 (29.2)	1
Child’s age (months)			
0–11	46 (68.7)	21 (31.3)	1
12–23	100 (76.9)	30 (23.1)	1.5 (0.8–2.9)
24–35	78 (67.8)	37 (32.2)	0.9 (0.5–1.8)
36–47	62 (68.1)	29 (31.9)	0.9 (0.5–1.9)
48–59	34 (69.4)	15 (30.6)	1.0 (0.5–2.3)
Child’s gender			
Male	151 (67.4)	73 (32.6)	0.7 (0.5–1.1)
Female	169 (74.1)	59 (25.9)	1

*1* Reference category

^a^Other ethnic groups included Welayta, Siltie, Gamo, Hadiya, and several others

**Table 3:** Bivariate analysis of enabling and need factors with health-seeking behavior in slums of Addis Ababa, October 2014–July 2015

Variables in blocks 1 and 2	Health-seeking		COR (95% CI)
	Sought care (%)	No care sought (%)	
Household monthly income			
50 US$ and above	105 (87.5)	15 (12.5)	3.8 (2.2–6.8)
Less than 50 US$^a^	215 (64.8)	117 (35.2)	1
Estimated walking time to reach health facilities			
Less than 15 min	89 (84.8)	16 (15.2)	2.8 (1.6–5.0)
15 min or more	231 (66.6)	116 (33.4)	1
Recognized danger signs^b^			
Three or more occurrences of watery/mucous stools in a day			
Yes	307 (70.9)	126 (29.1)	1.1 (0.4–3.0)
No	13 (68.4)	6 (31.6)	1
Fever			
Yes	271 (80.9)	64 (19.1)	5.9 (3.7–9.3)
No	49 (41.9)	68 (58.1)	1
Thirsty			
Yes	154 (84.2)	29 (15.8)	3.3 (2.1–5.3)
No	166 (61.7)	103 (38.3)	1
Vomiting			
Yes	201 (85.5)	34 (14.5)	4.9 (3.1–7.6)
No	119 (54.8)	98 (45.2)	1
Refused to eat/drink			
Yes	146 (74.1)	51 (25.9)	1.3 (0.9–2.0)
No	174 (68.2)	81 (31.8)	1
Number of recognized danger signs			
1	35 (41.2)	50 (58.8)	1
2	50 (49.5)	51 (50.5)	2.3 (1.2–4.3)
3	51 (73.9)	18 (26.1)	4.0 (2.0–8.1)
4 and above	184 (84.8)	33 (15.2)	8.0 (4.5–14.1)
Perceived cause of acute diarrhea			
Evil eye	19 (63.3)	11 (36.7)	1
Teething	51 (73.9)	18 (26.1)	1.1 (0.4–3.4))
Infection/weaning	22 (68.8)	10 (31.2)	1.8 (0.7–4.9)
Drinking contaminated water	46 (78.0)	13 (22.0)	1.4 (0.5–4.3)
Poor hygiene and sanitation	168 (70.3)	71 (29.7)	2.3 (0.8–6.4)
No knowledge about the cause	14 (60.9)	9 (39.1)	1.5 (0.6–3.7)

*1* Reference category

^a^The average exchange rate of 1 US$ (United States Dollars) was 20.43 ETB (Ethiopian birr) from October 2014–July 2015

^b^Danger signs mean the mothers/caregivers recognition of the severity of illness and the need for care

In multivariable analysis, predisposing factors of literacy of mothers/caregivers (adjusted odds ratio (AOR) = 2.4; 95% CI 1.4–4.1) and occupation of mothers/caregivers (not being a housewife) (AOR = 2.6; 95% CI 1.5–4.6) were significantly associated with health-seeking behavior. Among enabling factors, having household monthly income of 50 US$ and above (AOR = 2.9; 95% CI 1.5–5.6) and availability of nearest health facilities within 15 min walking distance (AOR = 3.3; 95% CI 1.7–6.6) significantly increased the odds of health-seeking behavior. Of the need factors, fever (AOR = 4.3; 95% CI 2.4–7.6) and vomiting (AOR = 3.3; 95% CI 1.8–5.9) as danger signs of severity of illness were significantly associated with health-seeking behavior (Table 4).

**Table 4:** Factors associated with health-seeking behavior in the multivariable logistic regression analysis

Variables	Model I	Model II	Model III	Final model
	AOR (95% CI)	AOR (95% CI)	AOR (95% CI)	AOR (95% CI)
Mothers/caregivers education				
Literate	3.1 (1.9–4.8)*			2.4 (1.4–4.1)*
Illiterate	1			1
Mothers/caregivers occupation				
Not housewives	1.7 (1.1–2.8)*			2.6 (1.5–4.6)*
Housewives	1			1
Household size				
2–5 persons	0.8 (0.5–1.2)			
6 or more persons	1			
Child’s age (months)				
0–11	1			1
12–23	1.6 (0.8–3.1)**			1.5 (0.7–3.4)
24–35	0.9 (0.5–1.8)			0.8 (0.3–1.7)
36–47	0.9 (0.4–1.8)			0.6 (0.3–1.5)
48–59	1.2 (0.5–2.6)			0.7 (0.3–1.9)
Child’s gender				
Male	0.8 (0.5–1.5)			
Female	1			
Time of walking to the nearest health facility (minutes)				
Less than 15 min		2.9 (2.2–7.3)*		3.3 (1.7–6.6)*
15 min or more		1		1
Household monthly income				
50 US$ and above		4.0 (2.2–7.3)*		2.9 (1.5–5.6)*
Less than 50 US$		1		1
Recognized danger signs				
Thirsty			1	1
Fever			3.2 (1.1–9.3)*	4.3 (2.4–7.6)*
Vomiting			2.9 (1.2–6.8)*	3.3 (1.8–5.9)*
Refused to eat/drink			0.5 (0.2–1.3)**	0.7 (0.4–1.2)
Number of recognized danger signs				
1			1	
2			1.1 (0.4–3.4)	
3			1.3 (0.2–7.7)	
4 and above			1.5 (0.1–18.7)	
Perceived cause of acute diarrhea				
Evil eye			1	1
Teething			1.2 (0.3–4.2)	1.1 (0.3–4.2)
Infection/weaning			1.3 (0.4–3.9)	1.5 (0.4–5.3)
Drinking contaminated water			1.2 (0.3–4.0)	1.2 (0.3–4.6)
Poor hygiene and sanitation			2.4 (0.7–7.5)**	2.5 (0.7–8.8)
Do not know			1.5 (0.5–3.9)	1.7 (0.6–5.0)

*1* Reference category

*Statistically significant at *p* < 0.05; **significant at *p* < 0.3 and included into the final model

### Utilization of health facilities

Among the 256 acute diarrheal under-five children taken to health facilities, 197 (76.9%) were seen at government health facilities, 46 (18.0%) at private health facilities, and 13 (5.1%) by informal health facilities (traditional healers). Among mothers/caregivers who preferred government health facilities, most did so due to the lower treatment cost 173 (87.8%). For those who preferred private health facilities, allegedly good quality of treatment was the main reason reported by 40 (86.9%) (Table 5). Among mothers/caregivers who sought treatment by consulting private and/or government health facilities, 182 (40.3%) were given home-recommended fluids (HRF) and 180 (39.8%) obtained oral rehydration salt (ORS). Only 54 (11.9%) were given ORS plus zinc supplementation at health facilities (Table 6).

**Table 5:** Health facilities utilization and reasons for seeking or not seeking care at health facilities in slums of Addis Ababa, October 2014–July 2015

Variables	Number (*n*)	Percentage (%)	95% CI
Reasons for not seeking care after recognizing acute diarrhea (N = 132)^a^			
Lack of money	104	78.8	70.9–85.0
Long distance to the health facilities	25	18.9	13.1–26.6
Illness was not serious	90	68.2	59.6–75.6
Mothers/caregivers was busy	68	51.5	42.9–60.0
High treatment cost	24	18.2	12.4–25.8
New teething	9	6.8	3.5–12.7
Types of health facilities used (N = 256)			
Government health facilities	197	76.9	71.4–81.7
Private health facilities	46	18.0	13.7–23.2
Informal health facilities	13	5.1	2.9–8.6
Types of government health facilities (N = 197)			
Health center	185	93.9	89.6–96.5
Hospital	12	6.1	3.4–10.4
Types of private health facilities (N = 46)			
Clinics^b^	28	60.9	45.7–74.2
Pharmacies or drug vendors	12	26.1	15.1–41.1
Hospital	6	13.0	5.8–26.8
Reasons for seeking care at government health facilities (N = 197)^a^			
Low cost of treatment	173	87.8	82.4–91.7
Good treatment	41	20.8	15.7–27.1
Near to home	120	60.9	53.8–67.5
Illness was serious	83	42.1	35.4–49.2
Treatment obtained within a short period of time	40	20.3	15.2–26.6
Reasons for seeking care at private health facilities (N = 46)^a^			
Good treatment	40	86.9	73.2–94.2
Near to home	23	50.0	35.4–64.6
Illness was serious	31	67.4	52.1–79.7
Treatment obtained within a short period of time	16	34.8	22.1–50.0
Reasons for seeking care at informal health facilities (N = 13)^a^			
Low cost of treatment	10	76.9	42.8–93.7
Good treatment quality	7	53.8	24.8–80.4
Near to home	8	61.5	30.5–85.3
Illness was serious	10	76.9	42.8–93.7
Treatment obtained within a short period of time	7	53.8	24.8–80.5

N Denominator

^a^There were multiple responses from the study participants

^b^Lower, medium, and/or higher clinics

**Table 6:** Types of treatment given and feeding status for acute diarrheal under-five children in slums of Addis Ababa, October 2014–July 2015

Variables	Number (*n*)	Percentage (%)
Treatment sought only by consulting private and/or government health facilities (N = 452)^a^ ^,b^		
ORS	180	39.8
HRF	182	40.3
ORS plus zinc supplementation	54	11.9
Not any treatment given	6	1.3
Estimated amount of food given compared to normal (N = 423)^c^		
More	24	5.7
Same as usual	59	13.9
Somewhat less	157	37.1
Much less	143	33.8
No food given	27	6.4
Do not know	13	3.1
Estimated amount of liquid given compared to normal (N = 423)^c^		
More	85	20.1
Same as usual	103	24.3
Somewhat less	120	28.4
Much less	87	20.6
No liquid given	15	3.5
Do not know	13	3.1

N Denominator

^a^There were multiple responses from the study participants

^b^Did not include the treatment without consulting health facilities, which might lower the proportion of the treatment sought

^c^Includes all mothers/caregivers who consulted health facilities to seek care and sought care without consulting health facilities and who did not intentionally provided weaning feeding/liquid to their children for treating acute diarrhea

### Weaning practices

One third 143 (33.8%) of the acute diarrheal under-five-year-old children were given weaning food much less than normal. Somewhat reduced amounts of liquids were reportedly given by 120 (28.4%) and greatly reduced amounts by 87 (20.6%) mothers/caregivers either with or without consulting health facilities (Table 6).

## Discussion

This community-based cross-sectional household study examined health facilities utilization and predictors of health-seeking behavior. Our main finding is that almost two thirds of under-five children with acute diarrhea sought care either at home or health facilities and one third did not receive any care. Out of those who sought care at health facilities, utilization of government health facilities was preferred by the majority, but prompt care within 24 h after recognizing acute diarrhea was low. Mothers/caregivers literacy, occupation, household monthly income, availability of health facilities within 15 min walking distance, and recognizing danger signs of fever and vomiting were significantly associated with health-seeking behavior.

Of the one third of mothers/caregivers who did not seek treatment for acute diarrhea reportedly gave weaning food and provided liquid for their children at home in different amounts compared to usual patterns. They did not seek care and continued weaning feedings, not with the intention of treating acute diarrhea. This might be due to mothers/caregivers expecting spontaneous recovery without treatment, lack of knowledge about the severity of the illness, failure to recognize that the child was with diarrhea, or the common perception that diarrhea was due to new teething. Similar findings in two slums of Nairobi, Kenya, indicated that 35% of mothers/caregivers did not seek any care [30]. A study in Niger also revealed that one third of acute diarrheal under-five-year-old children were not taken to health facilities [31]. Another study, in Kolkata slums in India, reported that educated mothers/caregivers sought care at health facilities and home [32].

Low household income was a major deterrent to urban mothers/caregivers to seek prompt care in Western Nepal [24]. We also found that more than half (56.6%) of mothers/caregivers sought care at health facilities, slightly below the rate reported by the 2016 Ethiopia Demographic Health Survey result for Addis Ababa [33]. Moreover, availability of health facilities within 15 min walking distance was a predictor for health-seeking behavior. Some studies pointed out that time needed to reach health facilities influences health-seeking behavior [34, 35]. An extensive study in Ethiopia reported that utilization of different types of government and private health facilities declined with distance from patient homes [36].

Severity of illness also increased seeking care in health facilities [23]. Our data also show that recognizing the severity of illness by danger signs of fever and vomiting increases the likelihood of seeking care, similar to other studies [24, 37]. Recognizing the danger signs and willingness to seek care tends to be associated with higher educational status and better household income. Contrary to our findings, some studies reported that perceived severity of illness was not a reliable predictor of care seeking by mothers/caregivers [38, 39]. However, an increasing body of empirical evidence indicates that children in low and middle income countries were more likely to receive care if their mothers/caregivers perceived illness to be serious [24, 37, 40–46]. We found that perceived cause of acute diarrhea was not a predictor for health-seeking, corroborating several other studies [24, 40, 43, 44].

Mothers/caregivers utilized government health facilities mainly due to low cost of treatment and private health facilities because of their allegedly higher quality of treatment. In contrast with our findings, Sreeramareddy et al. [23] in India reported that private health facilities were the first choice for treating childhood illnesses. Several studies found that high cost of treatment for childhood illnesses at health facilities were the most common deterrent to health facility utilization in rural and urban communities [47–49] and that service quality predicts choice of health facilities [50]. These results, as well as those from other studies, suggest that choice of utilization of health facilities depend on treatment cost and quality of health service. Promoting and supporting public health centers improves access for low-income populations [51].

ORS plus zinc supplementation for acute diarrheal treatment was used relatively infrequently by our study population, whereas ORS and HRF were used by four times more of the mothers/caregivers than that of ORS plus zinc supplementation. The low use of ORS plus zinc supplementation in our study might be due to lack of advocacy by government health offices, health extension workers, non-governmental organizations, and civic societies who are interested in promoting ORS plus zinc supplementations. The utilization rates of ORS is slightly lower than the aggregated data of the 2011 Demographic Health Survey report of fluid from ORS packets (43.4%) [17] and lower than the 2016 Demographic Health Survey report of fluid from ORS packets (55.8%) in Addis Ababa [33]. This indicates inequitable use of ORS between the whole Addis Ababa and the slums of Addis Ababa, which might be due to lower socioeconomic status of slum residents and lack of proximity of health facilities for slum mothers/caregivers. In contrary with our findings, a similar study in Niger by Page et al. [31] found that ORS was used by almost three fourth of mothers/caregivers for treating diarrhea.

### Strength and limitations

By adapting predisposing, enabling, and need factors of Andersen behavioral model, we were able to identify the most important predictors for health-seeking behavior for acute diarrheal under-five children in slums of Addis Ababa. As a limitation, our conceptual framework did not consider quality of care sought and several community level variables which might play a role in determining care seeking. Addressing disparities in health access should ensure that everyone has access to high-quality care [52]. Our data analysis did not consider utilization of health facilities as an outcome variable. Instead, we used health-seeking behavior as an outcome. However, we analyzed descriptive statistics for different types of health facilities utilized and underlying reasons for the choices, which explain the health-seeking behavior in health facilities.

## Conclusions

Our findings show health facilities becoming a better choice to seek care than seeking care at home. The results suggest that upgrading of slums as part of urban development, particularly infrastructure development including the construction of health facilities within slums, and raising the socioeconomic level of the slum populations can increase access to care for mothers/caregivers with acute diarrheal under-five children. Rates of prompt and effective treatment, both at home and health facilities, may be further increased by health promotion programs targeting illiterate mothers/caregivers and poor households. The Urban Safety Net Program, enacted by the Ministry of Urban Development and Housing of Ethiopia in January 2017, is seeking to raise the socioeconomic status and livelihoods of poor residents in Addis Ababa and may thus foster conditions necessary for successful interventions, and the impact of this program for increasing health-seeking behavior and health facilities utilization should be studied. Further studies that integrate household, community, and health facility information pertaining to predisposing, enabling, and need factors at the household, health facility, and community levels may provide comprehensive assessments of opportunities and constraints in promoting health-seeking and health facility utilization for treating acute diarrheal under-five children in Addis Ababa slums.

## Abbreviations

AOR: Adjusted odds ratio
CI: Confidence interval
COR: Crude odds ratio
HRF: Home-recommended fluids
ORS: Oral rehydration salt
SD: Standard deviations
US$: United States Dollars
WHO: World Health Organization

## References

[1] Wardlaw T, Salama P, Brocklehurst C, Chopra M, Mason E (2010). Diarrhoea: why children are still dying and what can be done. Lancet.

[2] UNICEF and WHO (2009). Diarrhoea: why children are still dying and what can be done. World Health Organization.

[3] Podewils L, Mintz E, Nataro J, Arashar U (2004). Acute infectious diarrhoea among children in developing countries. Semin Pediatr Infect Dis.

[4] Pelto P, Pelto G (1992). Developing applied medical anthropology in third world countries: problems and actions. Soc Sci Med.

[5] WHO and UNICEF (2012). Counsel the mother: integrated management of childhood illnesses (IMCI). Divsion of child health and development. IMCI guidelines.

[6] Claeson M, Waldman RJ (2000). The evolution of child health programmes in developing countries: from targeting diseases to targeting people. Bull World Health Organ.

[7] Andersen R (1968). A behavioral model of families’ use of health services.

[8] Andersen R, Newman JF (1973). Societal and individual determinants of medical care utilization in the United States. Milbank Mem Fund Q Health Soc.

[9] Andersen R, Rice T, Kominski G (2001). Changing the US health care system: Key issues in health services, policy, and management.

[10] Azage M, Kumie A, Worku A, Bagtzoglou AC. Childhood diarrhea in high and low hotspot districts of Amhara Region, Northwest Ethiopia: a multilevel modeling. J Health Popul Nutr. 2016;35(13). doi:10.1186/s41043-016-0052-2. 10.1186/s41043-016-0052-2 PMC5025988 27184552

[11] Babitsch B, GohI D, Von LT. Re-revisiting Andersen’s behavioral model of health services use: A systematic review of studies from 1998-2011. GMS Psychosoc Med. 2012;9. doi:10.3205/psm000089. 10.3205/psm000089 PMC3488807 23133505

[12] Ghosh R. Child mortality in India: A complex situation. World J Pediatr. 2012;8(1):11–8. 10.1007/s12519-012-0331-y 22282378

[13] Srivastava NM, Awasthi S, Agarwal GG. Care seeking behavior and out-of-pocket expenditure for sick newborns among urban poor in Lucknow, Northern India: A prospective follow up study. BMC Health Serv Res. 2009;9(61). doi:10.1186/1472-6963-9-61. 10.1186/1472-6963-9-61 PMC2676263 19341473

[14] Thind A (2004). Health service use by children in rural Bihar. J Trop Pediatr.

[15] Fosu GB (1994). Childhood morbidity and health services utilization: cross-national comparisons of user-related factors from DHS data. Soc Sci Med.

[16] Chowdhury ME, Ronsmans C, Killewo J, Anwar I, Gausia K, Das-Gupta S (2006). Equity in use of home-based or facility-based skilled obstetric care in rural Bangladesh: an observational study. Lancet.

[17] Ethiopia Demographic and Health Survey: Ethiopia Demographic and Health Survey 2011. Addis Ababa, Ethiopia, and Calverton, Maryland, USA. Central Statistical Agency [Ethiopia] and ORC Macro; 2012.

[18] Central Statistical Agency. Population projection of Ethiopia for all regions, at *woreda* level from 2014-2017. Addis Ababa: Central Statistical Agency (CSA); 2013.

[19] Federal Ministry of Health of Ethiopia (2005). National strategy for child survival in Ethiopia.

[20] Kelsey J, Whittemore A, Evans A, Thompson W (1996). Methods of sampling and estimation of sample size. Methods in observational epidemiology.

[21] World Health Organization (2005). The treatment of diarrhea. A manual for physicians and other senior health workers.

[22] Farthing M, Salam M, Lindberg G, Dite P, Khalit I, Lindo-Salazar E, et al. World Gastroenterology Organization Global Guidelines. Acute diarrhea in adults and children: a global perspective. World Gastroenterology Organization. 2012;1–24. http://www.worldgastroenterology.org/UserFiles/file/guidelines/acute-diarrhea-english-2012.pdf. Accessed 10 May 2014.

[23] Sreeramareddy C, Sathyanarayana T, Kumar H (2012). Utilization of health care services for childhood morbidity and associated factors in India: a national cross-sectional household survey. PLoS One.

[24] Sreeramareddy CT, Shankar RP, Sreekumaran BV, Subba SH, Joshi HS, Ramachandran U. Care seeking behaviour for childhood illness-a questionnaire survey in western Nepal. BMC Int Health Hum Rights. 2006;6(7). doi:10.1186/1472-698X-6-7. 10.1186/1472-698X-6-7 PMC1543657 16719911

[25] StataCorp (2015). Statistical software.

[26] Kirkwood BR, Sterne JA, Kirkwood BR, Sterne JAC (2003). Medical statistics: chapter 29: regression modelling. Essential medical statistics.

[27] McNamee R (2003). Confounding and confounders. Occup Environ Med.

[28] Bursac Z, Gauss CH, Williams DK, Hosmer DW. Purposeful selection of variables in logistic regression. Source Code Biol Med. 2008;3(17). doi:10.1186/1751-0473-3-17. 10.1186/1751-0473-3-17 PMC2633005 19087314

[29] Hosmer J, David W, Lemeshow S, Sturdivant RX (2013). Applied logistic regression.

[30] Mukiira C, Ibisomi L (2013). Health care seeking practices of caregivers of children under-five with diarrhea in two informal settlements in Nairobi, Kenya. J Child Health Care.

[31] Page AL, Hustache S, Luquero FJ, Djibo A, Manzo ML, Grais RF. Health care seeking behavior for diarrhoea in children under-five in rural Niger: Results of a cross-sectional survey. BMC Public Health. 2011;11(389). doi:10.1186/1471-2458-11-389. 10.1186/1471-2458-11-389 PMC3121637 21612640

[32] Manna B, Nasrin D, Kanungo S, Roy S, Ramamurthy T, Kotloff KL (2013). Determinants of health care seeking for diarrhoeal illness in young children in urban slums of Kolkata, India. Am J Trop Med Hyg.

[33] Ethiopia Demographic and Health Survey. Ethiopia Demographic and Health Survey 2016: key indicators report. Addis Ababa: Central Statistical Agency (CSA) [Ethiopia] and ICF; 2016.

[34] NoorAli R, Luby S, Rahbar M (1999). Does use of a government service depend on distance from the health facility?. Health Policy Plan.

[35] Okwaraji YB, Cousens S, Berhane Y, Mulholland K, Edmond K (2012). Effect of geographical access to health facilities on child mortality in rural Ethiopia: a community based cross-sectional study. PLoS One.

[36] Kloos H (1990). Utilization of selected hospitals, health centres and health stations in central, southern and western Ethiopia. Soc Sci Med.

[37] Taffa N, Chepngeno G (2005). Determinants of health care seeking for childhood illnesses in Nairobi slums. Trop Med Int Health.

[38] Herman E, Black RE, Wahba S, Khallaf N (1994). Developing strategies to encourage appropriate care‐seeking for children with acute respiratory infections: an example from Egypt. Int J Health Plann Manage.

[39] Hill Z, Kendall C, Arthur P, Kirkwood B, Adjei E (2003). Recognizing childhood illnesses and their traditional explanations: exploring options for care-seeking interventions in the context of the IMCI strategy in rural Ghana. Trop Med Int Health.

[40] Yoder PS, Hornik RC (1994). Perceptions of severity of diarrhoea and treatment choice: a comparative study of HealthCom sites. J Trop Med Hyg.

[41] Goldman N, Heuveline P (2000). Health-seeking behaviour for child illness in Guatemala. Trop Med Int Health.

[42] Goldman N, Pebley A, Gragnolati M (2002). Choices about treatment for ARI and diarrhea in rural Guatemala. Soc Sci Med.

[43] Pillai RK, Williams SV, Glick HA, Polsky D, Berlin JA, Lowe RA (2003). Factors affecting decisions to seek treatment for sick children in Kerala, India. Soc Sci Med.

[44] Thind A (2005). Analysis of health services use for respiratory illness in Indonesian children: implications for policy. J Bioso Sci.

[45] De-Silva M, Wijekoon A, Hornik R, Martines J (2001). Care seeking in Sri Lanka: one possible explanation for low child mortality. Soc Sci Med.

[46] Gelaw YA, Gashaw AB, Kefyalew A. Effect of residence on mothers’ health care seeking behavior for common childhood illness in Northwest Ethiopia: a community based comparative cross-sectional study. BMC Res Notes. 2014;7(705). doi:10.1186/1756-0500-7-705. 10.1186/1756-0500-7-705 PMC4210615 25297952

[47] Tarimo D, Lwihula G, Minjas J, Bygbjerg I (2000). Mothers’ perceptions and knowledge on childhood malaria in the holo-endemic Kibaha district, Tanzania: implications for malaria control and the integrated managemnet of childhood illnesss (IMCI) strategy. Trop Med Int Health.

[48] Thind A, Andersen R (2003). Respiratory illness in the Dominican Republic: what are the predictors for health services utilization of young children?. Soc Sci Med.

[49] Thind A, Cruz A (2003). Determinants of children’s health service utilization in the Philippines. J Trop Pediatr.

[50] Andaleeb SS (2000). Public and private hospitals in Bangladesh: service quality and predictors of hospital choice. Health Policy Plan.

[51] Andersen RM, Yu H, Wyn R, Davidson PL, Brown ER, Teleki S (2002). Access to medical care for low-income persons: how do communities make a difference?. Med Care Res Rev.

[52] Derose KP, Gresenz CR, Ringel JS (2011). Understanding disparities in health care access and reducing them through a focus on public health. Health Aff.

[53] Council for International Organizations of Medical Sciences. International ethical guidelines for biomedical research involving human subjects. Bull Med Ethics. 2002;182:17–23. PMID:14983848. 14983848

