# Erratum to: Utilization of health facilities and predictors of health-seeking behavior for under-five children with acute diarrhea in slums of Addis Ababa, Ethiopia: a community-based cross-sectional study

**DOI:** 10.1186/s41043-017-0091-3

**Published:** 2017-05-04

**Authors:** Metadel Adane, Bezatu Mengistie, Worku Mulat, Helmut Kloos, Girmay Medhin

**Affiliations:** 1 Ethiopian Institute of Water Resources (EIWR), Addis Ababa University, Addis Ababa, Ethiopia; 2 College of Health and Medical Sciences, Haramaya University, Haramaya, Ethiopia; 3 Department of Civil and Environmental Engineering, University of Connecticut, Storrs, USA; 4 Department of Epidemiology and Biostatistics, University of California, San Francisco, USA; 5 Aklilu Lemma Institute of Pathobiology, Addis Ababa University, Addis Ababa, Ethiopia

## Erratum

Upon publication of the original article [1], it was noticed that the second sentence of Section Methods, sub-section study setting, ‘Three slum kebeles in Gullele Sub-City’s District (Woreda) 01 and four slum kebeles in Lideta Sub-City’s District 05 were included in the study’ was incorrectly given as ‘Four slum kebeles in Gullele Sub-City’s District (Woreda) 01 and three slum kebeles in Lideta Sub-City’s District 05 were included in the study’. This has now been acknowledged and corrected in this erratum.
